# OPDA Has Key Role in Regulating Plant Susceptibility to the Root-Knot Nematode *Meloidogyne hapla* in *Arabidopsis*

**DOI:** 10.3389/fpls.2016.01565

**Published:** 2016-10-24

**Authors:** Cynthia Gleason, Natthanon Leelarasamee, Dorothea Meldau, Ivo Feussner

**Affiliations:** ^1^Department of Plant Molecular Biology and Physiology, Georg August University - Albrecht von Haller InstituteGöttingen, Germany; ^2^Department of Plant Molecular Biology and Physiology, Georg August University - Göttingen Center for Molecular BiosciencesGöttingen, Germany; ^3^Department of Plant Biochemistry, Georg August University - Albrecht von Haller InstituteGöttingen, Germany; ^4^Department of Plant Biochemistry, Georg August University - Göttingen Center for Molecular BiosciencesGöttingen, Germany

**Keywords:** COI1, induced resistance, Jasmonic acid, nematodes, opda, plant defense, plant hormones

## Abstract

Jasmonic acid (JA) is a plant hormone that plays important roles in regulating plant defenses against necrotrophic pathogens and herbivorous insects, but the role of JA in mediating the plant responses to root-knot nematodes has been unclear. Here we show that an application of either methyl jasmonate (MeJA) or the JA-mimic coronatine (COR) on *Arabidopsis* significantly reduced the number of galls caused by the root-knot nematode *Meloidogyne hapla*. Interestingly, the MeJA-induced resistance was independent of the JA-receptor COI1 (CORONATINE INSENSITIVE 1). The MeJA-treated plants accumulated the JA precursor *cis*-(+)-12-oxo-phytodienoic acid (OPDA) in addition to JA/JA-Isoleucine, indicating a positive feedback loop in JA biosynthesis. Using mutants in the JA-biosynthetic pathway, we found that plants deficient in the biosynthesis of JA and OPDA were hyper-susceptible to *M. hapla.* However, the *opr3* mutant, which cannot convert OPDA to JA, exhibited wild-type levels of nematode galling. In addition, mutants in the JA-biosynthesis and perception which lie downstream of *opr3* also displayed wild-type levels of galling. The data put OPR3 (OPDA reductase 3) as the branch point between hyper-susceptibility and wild-type like levels of disease. Overall, the data suggests that the JA precursor, OPDA, plays a role in regulating plant defense against nematodes.

## Introduction

Root-knot nematodes (*Meloidogyne* sp.) are small soil-borne pathogens that can infect more than 5,000 different plant species and cause significant yield losses (Sasser and Freckman, 1987; Koenning et al., 1999; Trudgill and Blok, 2001). During plant infection, stage 2 juveniles (J2) penetrate the roots behind the root cap and migrate intercellularly into the root vasculature where they will eventually settle and form feeding sites. During feeding site formation, the nematode chooses between 2 and 12 plant cells to pierce with its feeding stylet and induce several rounds of cellular endoreduplication without cytokinesis; the resulting enlarged, multinucleate feedings cells are called giant cells (Williamson and Gleason, 2003; Gheysen and Mitchum, 2011; de Almeida Engler and Gheysen, 2013; Perry and Moens, 2013). The giant cells give the nematode the nutrients to provide the energy to complete its life cycle and the adult female will lay eggs in a gelatinous matrix on the outside of the root. While the giant cells are forming, the parenchyma cells that surround the giant cells also divide, and as a result, large root galls, also known as “root knots,” develop in the root systems. Root galling is one of the most obvious disease symptoms resulting from root-knot nematode infection, and it can reflect disease severity.

**FIGURE 1:**
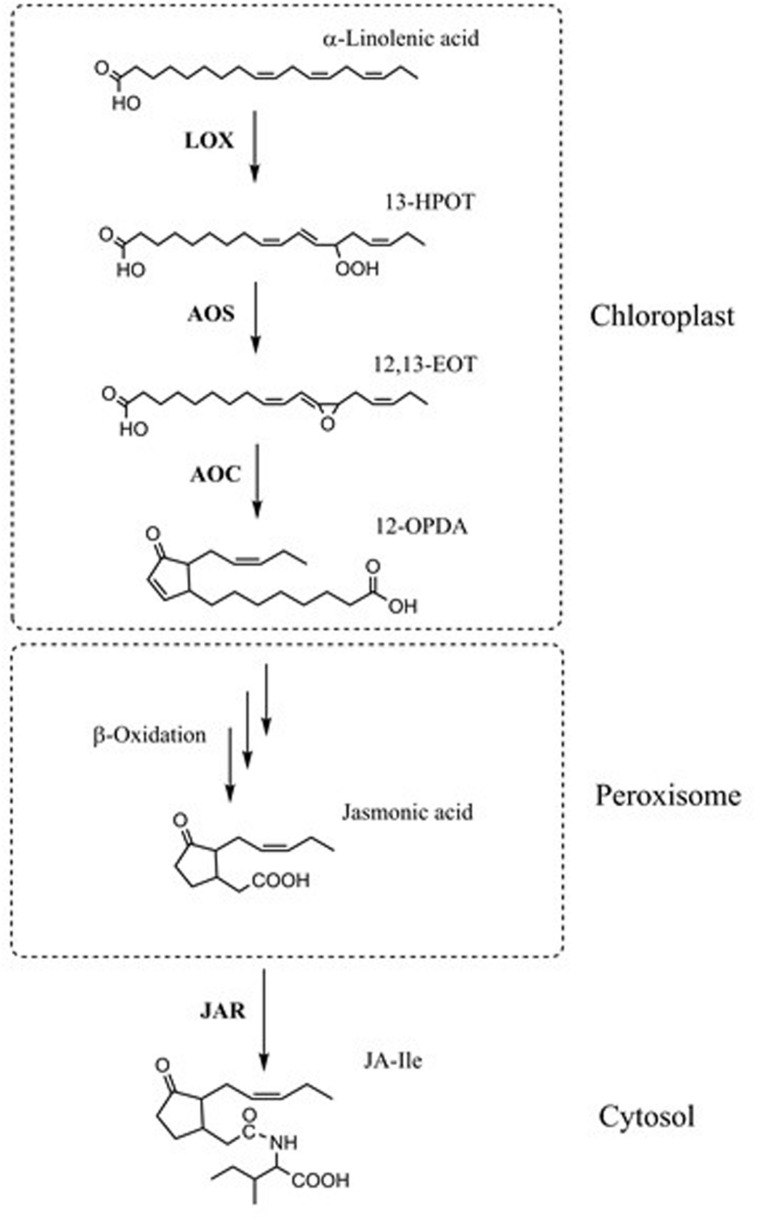
**Simplified JA biosynthetic pathway.** α-Linolenic acid is oxygenated by 13-lipoxygenases (LOXs). In the 13-LOX pathway, the product (13*S*)-hydroperoxy-octadecatrienoic acid (13-HPOT), is a substrate for allene oxide synthase (AOS). AOS converts 13-HPOT to (12,13*S*)-epoxyoctadecatrienoic acid (EOT). Allene oxide cyclase (AOC) converts EOT to *cis*-(+)-12-oxo-phytodienoic acid (12-OPDA). The OPDA is transported into peroxisome where it is converted to jasmonic acid (JA) by several rounds of β-oxidation. Jasmonic acid moves to the plant cytoplasm where it can be converted to (+)-7-*iso*-jasmonoyl-L-isoleucine (JA-Ile) by JAR1.

Jasmonic acid (JA) is an important plant hormone with roles in plant development and defense (Browse, 2005; Glazebrook, 2005; Shah, 2009; Wasternack and Hause, 2013; Heitz et al., 2016). JA is derived from polyunsaturated α-linolenic acid (18:3(*n*-3); *x*:*y*(*n*-*z*) denotes a fatty acid with *x* carbons and *y* double bonds in position *z* counting from the methyl end) and roughanic acid (16:3(*n*-3)), which are oxygenated by either 9-lipoxygenases (LOX1 and LOX5 in *Arabidopsis*) or 13-lipoxygenases (LOX2, LOX3, LOX4, and LOX6 in *Arabidopsis*) (Bannenberg et al., 2009). The 13-LOX makes 13-hydroperoxy-octadecatrienoic acid (13-HPOT) from 18:3(*n*-3) and 11-hydroperoxy-hexadecatrienoic acid (13-HPHT) from 16:3(*n*-3). Both are substrates for allene oxide synthase (AOS). The AOS products are converted to *cis*-(+)-12-oxo-phytodienoic acid (OPDA) or dinor-12-oxo-phytodienoic acid (dn-OPDA) by allene oxide cyclase (Stenzel et al., 2003). The cyclopentenones are then transported into the peroxisome where they are converted to cyclopentanones by the peroxisomal enzyme OPR3 (OPDA reductase 3). The CoA esters of the products of OPR3 are subjected to β-oxidation by acyl-CoA oxidase (ACX) enzymes, leading to JA (Stintzi and Browse, 2000). JA moves to the plant cytoplasm where it can be converted to many compounds, including (+)-7-iso-jasmonoyl-L-isoleucine (JA-Ile), which can bind to the JA-receptor COI1 in *Arabidopsis* (Fonseca et al., 2009) (**Figure 1**). The binding of JA-Ile to COI1 ultimately releases transcriptional repression of JA-responsive genes (Chini et al., 2007; Thines et al., 2007; Yan et al., 2007). Transcriptional profiling has shown that a majority of JA-responsive genes are COI1-dependent (Devoto et al., 2005; Taki et al., 2005).

Pathogens have evolved sophisticated strategies in order to avoid plant defenses. For example, the bacterium *Pseudomonas syringae* produces a toxin called coronatine (COR) (Feys et al., 1994). COR is a structural mimic of JA-Ile and it can interact with COI1 with even higher affinity than JA-Ile (Bender et al., 1999; Yan et al., 2009). *P. syringae* induces salicylic acid (SA)-mediated defense, but COR promotes bacterial virulence by taking advantage of the negative cross-talk between JA and SA. By mimicking JA, COR helps to abrogate the SA-mediated defenses against this bacterial pathogen. In addition, COR prevents stomatal closure which facilitates the invasion of *P. syringae* into the plant through these openings (Brooks et al., 2005; Cui et al., 2005; Melotto et al., 2006).

For plant-parasitic nematodes, there is no evidence that the nematode is making a JA-mimic like COR to facilitate infection. However, during the early stages of giant cell formation in *Arabidopsis*, defense gene expression is down-regulated relative to the expression in the un-infected tissue (Barcala et al., 2010). In *Medicago truncatula* giant cells, genes involved in the biosynthesis of JA and its derivatives are down-regulated (Damiani et al., 2012). Some JA-biosynthesis genes and JA-signaling responses are down-regulated during cyst nematode *Heterodera glycines* infection of susceptible soybean (Ithal et al., 2007a,b). During the early compatible interaction with rice, *Meloidogyne graminicola* suppresses defense gene expression, including the JA-responsive PR gene *JiOsPR10* (Nahar et al., 2011). These data would suggest that, in general, plant parasitic nematodes are actively downregulating defense gene expression, and in particular, suppressing the JA-mediated signaling pathways. Conversely, exogenous applications of MeJA and JA have been shown to activate nematode resistance in several crop plants (Soriano et al., 2004a,b; Cooper et al., 2005). In rice the MeJA-induced resistance correlated with enhanced expression of JA biosynthesis and defense genes (Nahar et al., 2011). It seems that upon MeJA-treatment, the nematode is no longer efficiently able to suppress or counteract plant defenses.

Although the data above would suggest that JA is involved in plant defense against nematodes, the role of JA is confounded by several reports suggesting that JA is required for nematode susceptibility. For example, Bhattarai et al. (2008) found that the JA perception mutant in tomato, *jai1*, had significantly reduced *M. incognita* infection. JAI1 in tomato is homologous to COI1 in *Arabidopsis*. Furthermore, work in maize has studied ZmLOX3 and found that it mediates suppression of biosynthesis of JA and SA (Gao et al., 2008). Therefore, *Zmlox3-4*, had increased levels of JA and SA, and yet the plants were more susceptible to root-knot nematodes compared to the wild type (Gao et al., 2008).

In this paper we show that by adding exogenous MeJA or the JA-mimic COR, we could induce resistance to the Northern root-knot nematode *Meloidogyne hapla* in *Arabidopsis*. We then utilized well-characterized JA signaling and biosynthesis mutants from *Arabidopsis* and found that COI1 is not required for nematode susceptibility and the MeJA-induced resistance is COI1-independent. Most importantly, our data provides evidence that the JA-precursor OPDA, not JA/JA-Ile, is a key defense signaling molecule involved in regulating plant susceptibility to nematodes.

## Materials and Methods

### Plant Genotypes

Seeds of *Arabidopsis* (*Arabidopsis thaliana*) accession Columbia (Col-0) (N1093) were used as wild-type controls when working with mutants in the Col-0 background. Mutants were *dde2-2* (von Malek et al., 2002), *coi1-t* (SALK 035548) from I. Heilmann (Martin-Luther University); *acx1/5* (Schilmiller et al., 2007), *fad3-2 fad7-2 fad8* (McConn and Browse, 1996), *tir1* (Salk CS3798) (Ruegger et al., 1998) and *cyp20–3* (SALK_001615C) (Dominguez-Solis et al., 2008). The Wassilewskija (WS) ecotype was used as the control with *opr3* (Stintzi and Browse, 2000) which is a mutant in the WS background.

### Nematode bioassays

To collect *Meloidogyne hapla* strain VW9 eggs, roots from infected tomato (*Solanum lycopersicum* Green Zebra) were mixed vigorously for 4 min in 10% commercial bleach for 5 min. The eggs were collected on a 25 μm sieve. The eggs were further surface sterilized in 10% bleach for 5 min and immediately rinsed one-time in water before another bleach step (10%, 5 min). The eggs then were washed three times with sterile H_2_O and then re-suspended in 5 ml water with 1% SDS and 2% Plant Preservative Mixture (Plant Cell Technology). Freshly hatched J2 were collected as described (Gleason et al., 2008).

*Arabidopsis* seeds were surface sterilized in 70% ethanol (EtOH) for 10 min, washed in 95% EtOH and allowed to air-dry. Seeds were placed MS media with 20% sucrose and placed in a growth chamber at 22°C/18°C, 80-100 μmol Photons/m^2^/s, 14 h light/10 h dark, 60% humidity. For the pre-treatment assays, plants were transferred to MS with or without 50 μM MeJA or 1 μM COR (final concentration). After 48 h in the growth chamber, plants were either collected for gene expression analysis or used in nematode assays. For gene expression analysis, root tissue was collected and frozen in liquid nitrogen. For the pre-treatment infection assays and for the *tir1* bioassays, five plants were transferred to modified KNOPs media in 100 square centimeter petri dishes and each plant was inoculated with 100 surface sterilized *M. hapla* J2. Inoculated plants were incubated in 14:10 h light: dark growth conditions. Galls were counted at 14 dpi (days post-inoculation).

We did not find any significant difference in nematode infections between Col-0 grown on MS and Col-0 grown modified KNOPs media (Sijmons et al., 1991) in our set-up (Supplementary Figure 1). Therefore, for all other nematode bioassays using JA biosynthetic mutants, 14 day old seedlings were grown on MS media, pH 5.7. The seedlings were in individual six well plates and each plant was inoculated with 100 *M. hapla*. The inoculated plants were kept in the dark at 22°C as this facilitates infection for root-knot nematode bioassays (Kyndt et al., 2016). Galls per plant were counted at 14 dpi. At 14 dpi, we observed galling, but the nematodes had not yet laid eggs. All experiments were repeated at least three times. All statistical analyses were performed using JMP software.

To monitor nematode penetration and infections, two week old seedlings were each inoculated with 100 *M. hapla* J2. At 4 dpi the plants were stained with acid fuchsin. These plants were placed into 50% commercial bleach solution for 2 min, rinsed with H_2_O and then placed into a boiling, 1/30 diluted, acid fuchsin staining solution (35 mg Acid fuchsin/100 mL) for 1 min. The stained plants were rinsed in H_2_O and observed under a stereo-microscope.

### Quantitative Reverse-Transcriptase Polymerase Chain Reaction (qRT-PCR)

Total RNA extraction and quantitative reverse-transcriptase polymerase chain reaction (qRT-PCR) analysis were performed as described (Fode et al., 2008). Calculations were done according to the 2^-Δ^*^C^*^T^ method (Livak and Schmittgen, 2001). *UBQ5* served as a reference (Kesarwani et al., 2007; Zander et al., 2010; Koster et al., 2012; Ralhan et al., 2012). Primers used to amplify and quantify the cDNA are as follows: VSP2 Forward 5′-CAAACTAAACAATAAACCATACCATAA-3′, VSP2 Reverse 5′-GCCAAGAGCAAGAGAAGTGA-3′. UBQ5 Forward 5′-GACGCTTCATCTCGTCC-3′, UBQ5 Reverse 5′-GTAAACGTAGGTGAGTCCA-3′.

### *coi1-t* Genotyping

Genomic DNA from leaves of infected plants was extracted DNeasy Plant Mini Kit (Quiagen, Valencia, CA, USA). This DNA was used as template for a three-primer polymerase chain reaction (PCR), to genotype the plants and determine which plants carry the T-insertion. A Standard PCR reaction was performed using reagents from the Advantage 2 PCR Enzyme System (Clontech). In the 10 μL reaction, the final primer concentrations were 0.5 μM and a final dNTP concentration was 0.2 μM. The reaction was conducted for 35 cycles at: 94°C 1 min., each cycle 94°C for 30 s., 68°C for 2:30 min. The primers used were as follows: COI1 Left Border (COI1 LB) 5′-TGGACCATATAAATTCATGCAGTCAACAAC-3′, COI1 Right Border (COI1 RB) 5′-CTGCAGTGTGTAACGATGCTCAAAAGTC-3′, and LBb1.3 5′-ATTTTGCCGATTTCGGAAC-3′. The products were separated on a 2% agarose gel. Wild-type plants will have a product only from the COI1 RB and COI1 LB primers (Mosblech et al., 2011).

### Plant Phytohormone Measurement by HPLC/MS

Eight day old Col-0 seedlings were transferred from MS media to MS media with or without 50 μM MeJA. The samples were collected after 48 hours of treatment and approximately 100 mg of root material (from approximately 500 seedlings) from three biological replicates were used. The extraction was performed as previously described in Matyash et al. (2008) with some modifications. Briefly, 100 mg frozen material was finely milled and extracted with 0.75 mL methanol and 2.5 mL MTBE (Methyl-tert-butylether) in the dark for 1 h at 4°C and constant shaking. To allow quantification deuterated standards (10 ng D6-JA, 30 ng D5-oPDA, 10 ng D3-JALeu, and 20 ng D5-IAA) were added to each sample. To enhance phase separation, 0.6 mL water was added to the sample before centrifugation. The upper phase was collected; the lower phase was re-extracted with 0.7 mL methanol/water (3:2.5, v/v) and 1.3 mL MTBE. Supernatants were combined and evaporated under a nitrogen stream. The residue was solved in 100 μL acetonitrile/water/acetic acid (20:80:0.1, v/v/v). During all extraction steps, direct light exposure was avoided to prevent photoisomerization. Further, between all steps samples were overlaid with argon to avoid autooxidation. Samples were subjected for phytohormone measurements on a HPLC-MS/MS as described by Iven et al. (2012).

Mass transitions (in Da) were as follows: 215/59 for D6-JA and 209/59 for JA; 296/170 for D5-oPDA, 263/165 for dinor-oPDA and 291/165 for cis-oPDA; 325/133 for D3-JA-Leu and 322/130 for JA-IleLeu; 179/135 for D5-IAA and 174/130 for IAA.

## Results

### Exogenous Application of MeJA and COR Elicits Nematode Resistance in *Arabidopsis*

**FIGURE 2:**
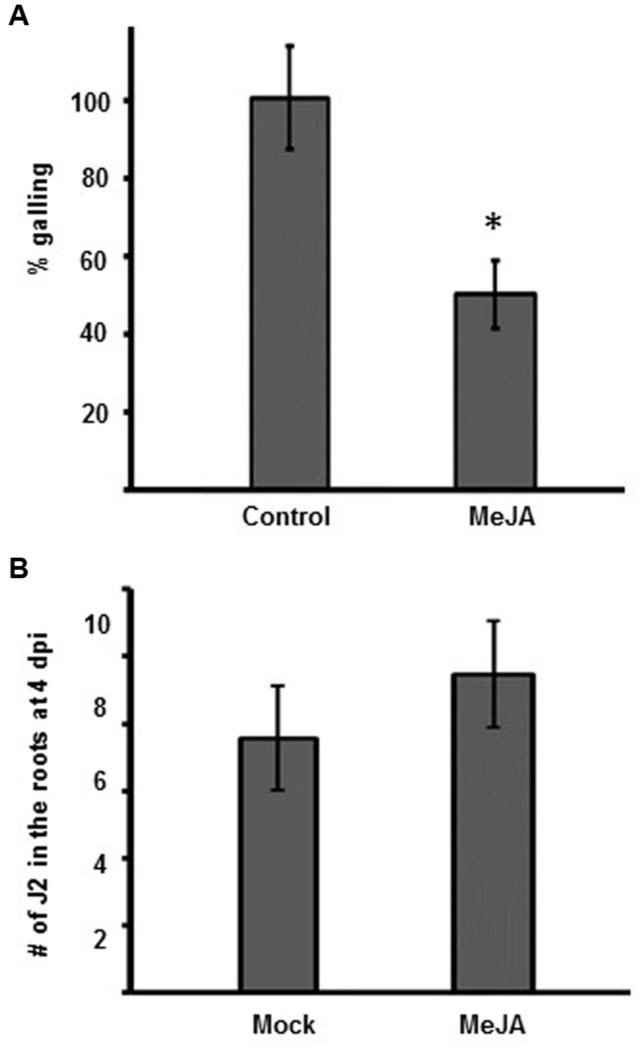
**Galling is reduced in *Arabidopsis* after MeJA. (A)** Relative galling at 14 dpi in Col-0 plants with or without pre-treatment with MeJA. The galling in untreated Col-0 (Control) was set to 100%. Values show the mean ± SE of three independent experiments. Untreated control *n* = 235, MeJA *n* = 155. Asterisk indicates a significant different between control and treatment group (Welch *t*-test ^∗^*p* < 0.005. **(B)** Number of J2 in the roots of plants, with or without 50 μM MeJA pre-treatment, at 4 dpi. Values are the means ± SE from three independent experiments (*n* = 15). No significant difference could be seen between mock and treatment group. Experiment repeated three times with similar results.

Exogenous application of MeJA has been shown to protect against *M. incognita* and *M. javanica* in several diverse crop plants and against *M. graminicola* in rice (Soriano et al., 2004a,b; Cooper et al., 2005; Nahar et al., 2011). The northern root-knot nematode *M. hapla* has a different host range to *M. incognita*. *M. hapla* also has a smaller genome and is found in more temperate climates. We wanted to determine if an exogenous application MeJA can induce resistance to *M. hapla* in *Arabidopsis*, and moreover, see if this induced resistance was specific to MeJA or if it could also be induced by the JA-analog COR. Therefore, we performed infection assays on plants pre-treated with MeJA or COR. Eight-day old Col-0 seedlings were transferred to Murashige–Skoog (MS) medium with or without 50 μM MeJA or 1 μM COR for 48 h. Plants treated with either MeJA or COR for 48 h showed enhanced expression of the JA-marker gene *VSP2* (VEGETATIVE STORAGE PROTEIN2) in both leaves and roots, compared to the untreated plants, indicating that JA-responses had been switched on by the treatments (Supplementary Figures 2 and 3). After 48 h of treatment, was no significant effect on root length by MeJA or COR; all seedlings were transferred onto media lacking MeJA and COR and subsequently inoculated with 100 *M. hapla* juveniles. At 14 dpi, the number of galls per plant was counted. The results show that exogenous MeJA treatment reduced galling by approximately 50% (**Figure 2B**; Supplementary Figure 2). The pre-treatment with COR also significantly reduced galling by 25% compared to galling in the un-treated plants (Supplementary Figure 3).

MeJA pre-treatment had a stronger effect on galling compared to the COR pre-treatment (**Figure 2A**; Supplementary Figure 3B). Therefore, we further focused on the effects of MeJA in nematode resistance. To determine whether MeJA treatment of Col-0 had an influence on nematode attraction or root penetration, Col-0 seedlings were treated with either water (mock) or MeJA (50 μM, final concentration) for 48 h prior to infection with RKN juveniles. At 4 days post infection, the roots were stained with acid fuchsin to visualize the nematodes that had successfully penetrated the roots. We only observed J2 in the roots at this time point. There was no significant difference in the number of J2 in the roots of mock or MeJA treated plants (**Figure 2B**).

### JA Perception is Not Required for *Arabidopsis* Nematode Susceptibility or the MeJA-Induced Nematode Resistance

**FIGURE 3:**
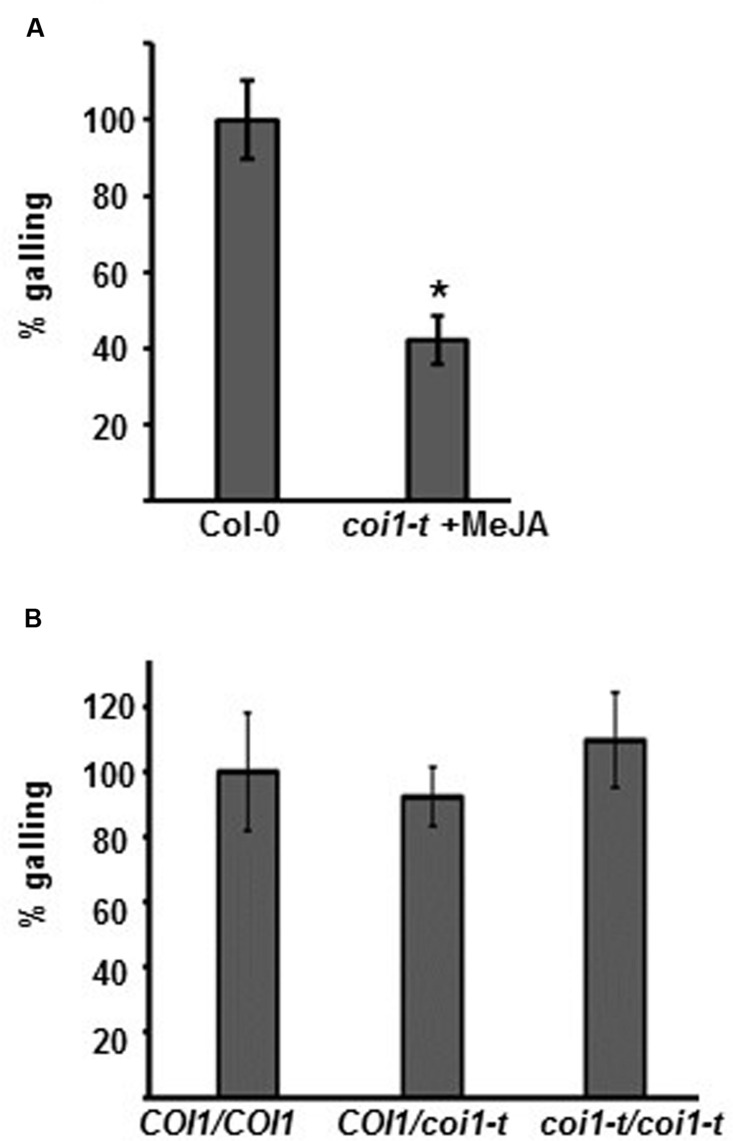
**Plant susceptibility to *Meloidogyne hapla* is COI1-independent. (A)** Col-0 seedlings, grown on MS without selection, and *coi1-t* seedlings, selected on MS + 50 μM MeJA media, were transferred to fresh MS plates and inoculated with 100 *M. hapla* J2 per plant. Galls per plant were counted at 14 dpi., and the galling in Col-0 was set to 100%. Bars represent mean values (±SE) from the combined results of three independent experiments (Col-0 *n* = 149, *coi1-t n* = 148). Asterisk indicates a significant different between mock and treatment group (Welch *t*-test ^∗^*p* < 0.05). **(B)** Ten-day-old seedlings on MS media were inoculated with 100 *M. hapla* J2 per plant. The severity of the infection was determined by counting galls at 14 dpi. The genotype of each plant was then determined by PCR (*COI1/COI1, COI1/coi1*, and *coi1/coi1*). The average galling in *COI1/COI1* (wild type) was set to 100%. Values represent the means ± SE from three independent experiments. (*n* = 35, 75, and 32, respectively).

Both the biologically active form of JA (JA-Ile) and COR have affinity for the JA-receptor COI1. We next studied whether *Arabidopsis coi1-t* mutant plants have altered *M. hapla* susceptibility. Because homozygous *coi1-t* plants are male sterile (Mosblech et al., 2011), the line is maintained with *coi1-t*/*COI1* heterozygous plants. To select homozygous *coi1-t*/*coi1-t* plants from the progeny of the *coi1-t*/*COI1* heterozygous line, seeds were germinated and grown for 10 days on MS media supplemented with 50 μM MeJA. Plants which showed no growth response to MeJA were assumed to be *coi1-t/coi1-t*. As a control, Col-0 plants were grown on MS without MeJA. Col-0 and the MeJA-insensitive *coi1-t/coi1-t* seedlings were then transferred to MS media. Two week old seedlings were inoculated with 100 *M. hapla*, and the number of galls per plant was counted at 14 dpi. The MeJA pre-treated *coi1-t* plants were significantly more resistant than the Col-0 seedlings (**Figure 3A**, Supplementary Figure 4).

Although *coi1-t* plants are MeJA insensitive, the MeJA pre-selection for homozygous *coi1-t* plants may have triggered JA-dependent, COI1-independent responses, leading to the induced resistance. To circumvent the MeJA pre-treatment, a segregating *coi1-t*/*COI1* line was grown on MS medium for 14 days, and the roots were inoculated with *M. hapla* juveniles. The number of galls per plant was counted at 14 dpi. Subsequently, the plants were collected for DNA extraction and the genotype at the *COI1* locus was determined by PCR for each plant. The *coi1-t*/*coi1-t* plants showed a similar number of galls as the *COI1/COI1* and *COI1*/*coi1-t* plants (**Figure 3B**; Supplementary Figure 4). This result indicates that COI1 is not significantly involved in the plants’ susceptibility to *M. hapla*. The resistance seen in the MeJA-treated plants *coi1-t* plants (**Figure 3A**) is due to the MeJA pre-treatment and not to the *coi1-t* genotype.

### JA Biosynthesis Mutants that Lie Upstream of OPR3 have Enhanced Nematode Susceptibility

**FIGURE 4:**
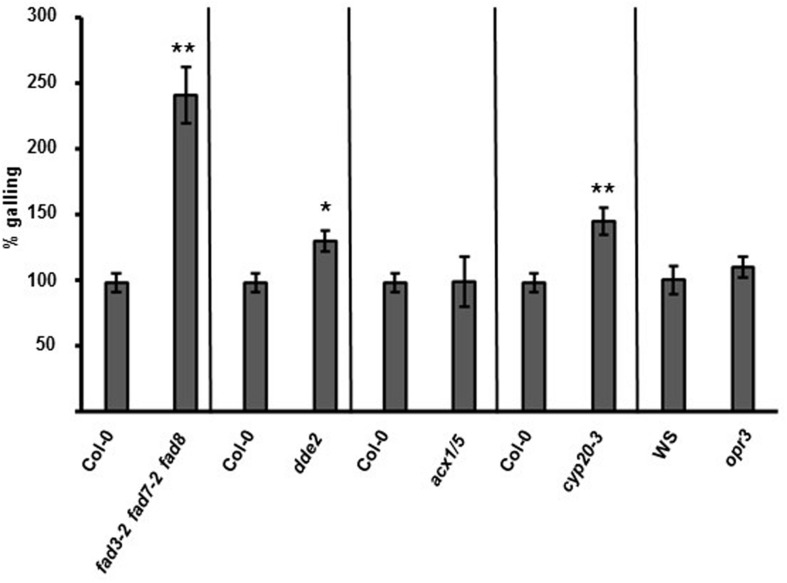
**Mutants in the JA biosynthetic pathway show differential susceptibility *M. hapla.* Fourteen-day-old seedlings on MS media were inoculated with 100 *M. hapla* J2 per plant and galls were counted at 14 dpi.** The average number of galls in Col-0 at 14 dpi was set to 100% and results are means ± SE combined from at least three independent experiments. Galling in each mutant was compared to galling in Col-0 (or the Wassilewskija ecotype (WS) for *opr3*). Asterisk indicates a significant difference between the mutant and control (Welch *t*-test ^∗^*p* < 0.0005, ^∗∗^*p* < 0.005). (*n* = 117, 39, 78, 47, 33, 33, and 29, respectively).

To investigate oxylipin signaling upstream of COI1, we studied the *Arabidopsis* fatty-acid desaturase triple mutant *fad3-2 fad7-2 fad8* that lacks 16:3(*n*-3) and 18:3(*n*-3) fatty acids (McConn and Browse, 1996). The *fad3-2 fad7-2 fad8* seedlings were inoculated with *M. hapla* juveniles and the number of galls per plant was counted 14 dpi. The *fad3-2 fad7-2 fad8* roots had significantly more galling than the wild-type roots (**Figure 4**; Supplementary Figure 5). In *Arabidopsis* the mutant *delayed dehiscence2* (*dde2-2*) is defective in AOS, and as a result, cannot produce JA (von Malek et al., 2002). The *dde2-2* plants exhibited enhanced nematode galling compared to the wild type (**Figure 4**; Supplementary Figure 5).

During the synthesis of oxylipins, AOS acts in a coupled reaction with AOC to produce a JA precursor OPDA. To determine if OPDA has a role in the plant–nematode interaction, an *Arabidopsis* mutant *opr3*, which is compromised in the conversion of OPDA to JA, was tested with nematodes. The *opr3* mutant is in the Wassilewskija ecotype (Stintzi and Browse, 2000). The *opr3* plants exhibited galling at levels similar to the Wassilewskija control (**Figure 4**; Supplementary Figure 5).

Next we tested the *acx1/5* mutant with *M. hapla*. The *acx1/5* mutant is defective in the β-oxidation steps downstream of OPR3 in the JA biosynthetic pathway (Schilmiller et al., 2007). At 14 dpi, the number of galls were counted in *acx1/5*, and the level of galling was to be similar to the Col-0 plants (**Figure 4**; Supplementary Figure 5).

### Plant OPDA Perception is Required to Maintain Wild-Type Levels of Nematode Galling

Our infection assays have shown that plants that cannot accumulate JA and OPDA are hyper-susceptible to nematodes. However, *opr3* plants, which cannot convert OPDA to JA, have wild-type levels of galling. Therefore, OPDA may have a key role in regulating plant susceptibility to nematodes. To test this hypothesis, we measured the *M. hapla*-susceptibility of a mutant in the recently identified OPDA receptor peptidyl-prolyl *cis*–*trans* isomerase3 (CYP20-3) (Park et al., 2013). Nematode bioassays were performed on *cyp20-3* seedlings, and at 14 dpi, there was more galling in the *cyp20-3* mutant compared to the wild-type plants (**Figure 4**; Supplementary Figure 5).

### Exogenous MeJA Treatments Increase the Content of JA-Precursors and JA in the Plant

**FIGURE 5:**
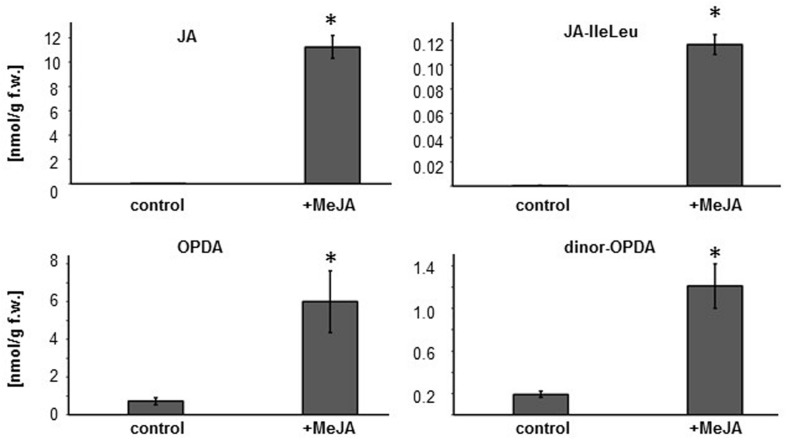
**MeJA treatment increases OPDA, dinor-OPDA, JA and JA-IleLeu concentrations in Col-0 roots.** Eight day old Col-0 seedlings were transferred onto MS media with or without 50 μM MeJA. After 48 h of treatment, approximately 100 mg of root was used for hormone measurements. Bars represent average nmol/gram fresh weight as measured by HPLC/MS. Error bar indicates standard error of mean (*n* = 3). Asterisk indicates a significant different between the mock and MeJA treated roots (student’s *t*-test ^∗^*p* < 0.05).

Since the evidence suggests that OPDA is regulating plant susceptibility to nematodes, we explored whether the MeJA-induced resistance in wild-type plants could be linked to elevated OPDA levels. Exogenous application of MeJA can induce the expression of JA biosynthesis genes (Turner et al., 2002; Wasternack et al., 2006), and this positive feedback loop in JA biosynthesis should result in enhanced OPDA synthesis. However, the effect of exogenous MeJA treatment on *Arabidopsis* hormone profile had not been fully investigated. Therefore, oxylipin profiling was carried out on roots of eight day old seedlings grown on MS media with or without MeJA treatment. Oxylipin profiling was conducted by using high-performance liquid chromatography/mass spectrometry (RP-HPLC/MS). We found that a 48 hr-MeJA treatment increased the content of OPDA, dinor-OPDA, JA and JA-IleLeu in the roots compared to the un-treated plants (**Figure 5**). In addition, the treatment did not affect the amount of auxin in the roots (data not shown).

### The Auxin Receptor TIR1 is Required for Nematode Susceptibility

**FIGURE 6:**
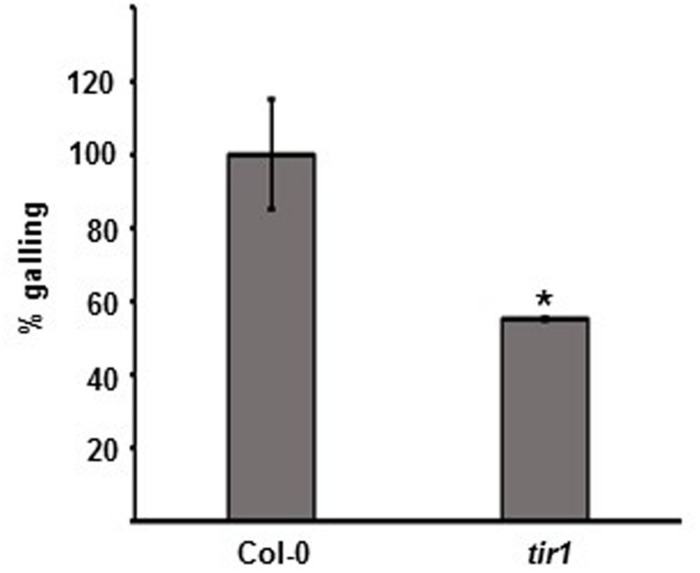
**The mutant in auxin perception *tir1* is more resistant to *M. hapla*.** Ten-day-old seedlings were inoculated with 100 *M. hapla* J2 per plant. Galls were counted at 14 dpi. Average galling in Col-0 was set to 100%. Values are means ± SE. Asterisk indicates a significant difference between Col-0 (*n* = 58) and *tir1* (*n* = 37) galling (Welch *t*-test ^∗^*p* < 0.01). Experiment repeated three times with similar results.

There is a body of evidence to suggest a positive interplay between oxylipin and auxin (Kazan and Manners, 2008). In addition, the JA receptor (COI1) and the auxin receptor (TIR1, TRANSPORT INHIBITOR RESPONSE 1) are both F box proteins that share structural similarity (Katsir et al., 2008). We next tested the requirement of the nuclear auxin receptor TIR1 in the plant-root-knot nematode interaction. After infection with *M. hapla, tir1* plants exhibited significantly less galling than the wild type, indicating that TIR1-mediated auxin signaling is important for nematode susceptibility (**Figure 6**; Supplementary Figure 6).

## Discussion

By using mutants in the JA biosynthetic pathway, we discovered a branch-point between hyper-susceptibility and wild type-levels of nematode disease symptoms. Mutants in enzymes upstream of OPR3 (*fad3-2 fad7-2 fad8* and *dde2-2*) were more susceptible to *M. hapla*, while plants with mutations in OPR3 (*opr3*), in downstream enzymes (*acx1/5*) or in JA perception (*coi1-t*) had wild-type galling. Based on our results, we postulate that other mutants in the JA biosynthetic pathway which lie upstream of OPR3 would be more susceptible to nematodes. Consistent with our hypothesis, Ozalvo et al. (2014) found that plants with a deficiency in lipoxygenase LOX4, an enzyme that catalyzes one of the first steps in JA biosynthesis, were more susceptible to nematodes than the control (Ozalvo et al., 2014). The rice *hebib*a mutant, which contains mutations in AOS, showed enhanced susceptibility to rice root-knot nematodes (Nahar et al., 2011), and mutants affected in JA biosynthesis (*dde2-2* and *lox6*) exhibited enhanced *Heterodera schachtii* female development (Kammerhofer et al., 2015). In conjunction with the mutant data, it was recently shown that blocking JA biosynthesis in rice by applying lipoxygenase (LOX) inhibitors significantly increased plant susceptibility toward root-knot nematodes (Nahar et al., 2011).

Our data show that plants that cannot produce JA or OPDA are more susceptible to nematodes, and this indicates JA and/or OPDA are the key defense molecule(s) in the plant-nematode interaction. Because *opr3* lacks the enzyme that reduces OPDA during JA biosynthesis, we could use *opr3* to differentiate between JA and OPDA-specific signaling (Sanders et al., 2000; Stintzi and Browse, 2000). The wild-type level of galling in the *opr3* mutant showed that in the absence of JA, OPDA is playing a critical role as a defense signal against nematodes. Interestingly, the validity of the *Arabidopsis opr3* work was recently called into question in a report by Chehab et al. (2011). The authors found that *opr3* plants produced full-length OPR3 transcripts upon Botrytis infection, revealing that *opr3* is not a null mutant, but a conditional, JA producing mutant (Chehab et al., 2011). This is in contrast to the previous work of Stintzi and Browse (2000) who had shown there was no induction of *OPR3* transcripts after fungal infection in the *opr3* mutant. The conflicting results may be due to differences in experimental conditions and/or pathogens that were tested (Botrytis vs. *A. brassicicola*). The nature of the *opr3* mutant continues to be investigated, but a separate study has now confirmed that that *Arabidopsis opr3* cannot produce OPR3 transcript upon *A. brassicicola* infection (Park et al., 2013). In addition, just as the *opr3* mutant was not compromised in resistance to a fungal pathogen, the RNAi - OPR3 tomatoes exhibited high levels of resistance to the larvae of the moth *Manduca sexta* (Bosch et al., 2014). These data support that *opr3* is useful mutant to study OPDA as a defense signaling molecule.

The increased susceptibility of the mutant in OPDA-perception (*cyp20-3*) also supports our hypothesis that OPDA has a major role in the plant–nematode interaction. The binding of OPDA to CYP20-3 alters cellular redox homeostasis and causes changes in gene expression during times of stress (Park et al., 2013). The increased galling in *cyp20-3* points to the importance of redox homeostasis in controlling the level of nematode susceptibility.

Although previous reports have shown MeJA can make plants more resistant to nematodes, the body of work has primarily focused on *M. incognita, M. javanica*, or *M. graminicola.* Here we show that exogenous MeJA application can induce resistance to the Northern root knot nematode *M. hapla*. MeJA likely has no toxic effects on root-knot nematodes since nematodes soaked in a MeJA solution can still infect and proliferate normally in tomato (Cooper et al., 2005). Alterations in ethylene signaling been shown to affect the attractiveness of a root to nematodes (Gao et al., 2008; Fudali et al., 2013). However, the MeJA-induce resistance was not the result of reduced root attraction or nematode penetration since at 4 dpi, there was no significant difference in the number of J2 in the root of control or MeJA treated plants (**Figure 2B**). Therefore, the reduction of galling after MeJA treatment was not due to changes in root attractiveness to nematodes or the nematode penetration of the roots. Considering the diversity in host-range, reproduction, and geographic distribution amongst species of root-knot nematodes, MeJA treatment elicits broad-spectrum nematode resistance, and downstream signaling pathways could be targets for manipulation in efforts improve plant resistance.

If OPDA is the main player in regulating plant resistance to nematodes, would exogenous MeJA treatment enhance nematode resistance? Our data supports the possibility that MeJA induces OPDA synthesis because the overall root content of OPDA and dn-OPDA increased after exogenous MeJA treatment in Col-0 plants. However, it is important to note that JA and JA-IleLeu also accumulated in the plants after the MeJA treatment. Therefore, we cannot completely rule out the contribution of JA/JA-Ile in the induced resistance response, and it is possible that the level of pathogen resistance may be due to the additive or synergistic effects of JA and/or other oxylipins (i.e., OPDA) (Park et al., 2013). Interestingly treating *Arabidopsis* with exogenous application of hexanoic acid primed the plants for production of OPDA and JA-Ile and increased plant resistance to Botrytis (Kravchuk et al., 2011). It would be interesting to investigate if hexanoic acid could also induce nematode resistance.

Although the effect was not as strong as with MeJA, the exogenous application of COR also reduced gall numbers. Since the JA-conjugate (JA-Ile) and COR can bind to the COI1-complex to activate JA-signaling, we initially thought that COI1 would be necessary for the induced resistance response. Surprisingly, the MeJA-treated COI1 seedlings also exhibited enhanced nematode resistance, indicating that the resistance was JA-dependent but COI1 independent. When we performed nematode bioassays in key JA biosynthetic mutants, OPDA and not JA played a key role in plant defenses against nematode. Based on the induced resistance response and the mutant data, we predict that in the absence of JA, OPDA is key for regulating plant immunity against nematodes. Since a pharmacological approach could not completely rule out the role of JA in the induced resistance response, we predict that the MeJA-induced resistance is likely due to the effects of both JA and OPDA acting through COI1-independent gene expression. The importance of JA in the induced response was re-iterated by the MeJA-induced resistance in *coi1t* plants. Future experiments will address if induced resistance can be linked to an overlapping set of genes which are regulated by both JA and OPDA in a COI1-independent manner.

To further clarify the role of COI1 in the plant-nematode interaction, we then tested *coi1-t* plants in a nematode bioassay without the MeJA pre-selection and found that these plants were just as susceptible as wild-type plants. This result is in contrast to that of Bhattarai et al. (2008), who found that in tomato, root-knot nematode susceptibility was dependent on COI1 mediated signaling. The differences in the requirement of COI1 may be the result of the different plant species used (tomato vs. *Arabidopsis*). In addition, the heterozygous population of the tomato *jai1* mutant had initially been screened for MeJA sensitivity (Bhattarai et al., 2008). A recent report found that MeJA treatment could induce nematode resistance for at least 1 week after the foliar application in tomato plants (Fujimoto et al., 2011), and thus, MeJA treatment may have a long lasting effects on plant responses to nematodes.

Although the focus of this study was on JA and members of the jasmonate family, hormone crosstalk plays an important role in tailoring these responses depending on the type of pathogen or stress encountered (Verhage et al., 2010; Robert-Seilaniantz et al., 2011). The cross talk between JA and auxin is generally positive, but a recent report found that when roots are exposed to high exogenous JA concentrations (50 μM), accumulation of the polar auxin transporter PIN2 on the plasma membrane is reduced, and by affecting the abundance of PIN proteins at the plasma membrane, JA may have a repressive role in auxin transport (Kazan and Manners, 2008; Sun et al., 2011). Interestingly, polar auxin transport (PAT) via specific PIN auxin influx carriers is crucial for the initiation and formation of the cyst nematode feeding site (Grunewald et al., 2009). For root-knot nematodes, there is a local accumulation of auxin at the nematode feeding sites and auxin responsive genes are turned-on in the gall tissue (Karczmarek et al., 2004; Grunewald et al., 2008). In addition, a recent report has shown that auxin import into root-knot nematode feeding sites is important for feeding site development and expansion (Kyndt et al., 2016). Perception of a local endogenous auxin accumulation in the cells would require the plant’s auxin receptor TIR1, an F-box protein that is part of the SKP1–Cullin–F-box protein complex (SCF^TIR1^) (Ruegger et al., 1998; Gray et al., 2001). The auxin receptor TIR1 is homologous to COI1; nevertheless, we found that while COI1 was not needed for nematode susceptibility, TIR1 was required for full nematode susceptibility as the *tir1* plants were more resistant to nematodes. Additional work needs to be performed to draw links between JA, OPDA, and auxin signaling in the plant-nematode interaction. However, in a recent paper looking at *Sclerotinia sclerotiorum*-infected *Arabidopsis*, there was partial overlap in the defense genes induced by *S. sclerotiorum* and those induced by exogenous OPDA treatment (Stotz et al., 2011). Most of these overlapping genes were expressed in a COI1-independent manner, and interestingly, the COI1-independent defenses against *S. sclerotiorum* were regulated by auxin signaling via ARF2 (Auxin Response Factor 2) (Stotz et al., 2011).

## Author Contributions

CG and IF designed the study; CG, NL, and DM performed the experiments. CG wrote the manuscript.

## Conflict of Interest Statement

The authors declare that the research was conducted in the absence of any commercial or financial relationships that could be construed as a potential conflict of interest.
