# Supplementary figures and images for: OPDA Has Key Role in Regulating Plant Susceptibility to the Root-Knot Nematode Meloidogyne hapla in Arabidopsis
Source: Front Plant Sci. 2016 Oct 24;7:1565. doi: 10.3389/fpls.2016.01565 (PMC5075541; doi:10.3389/fpls.2016.01565)

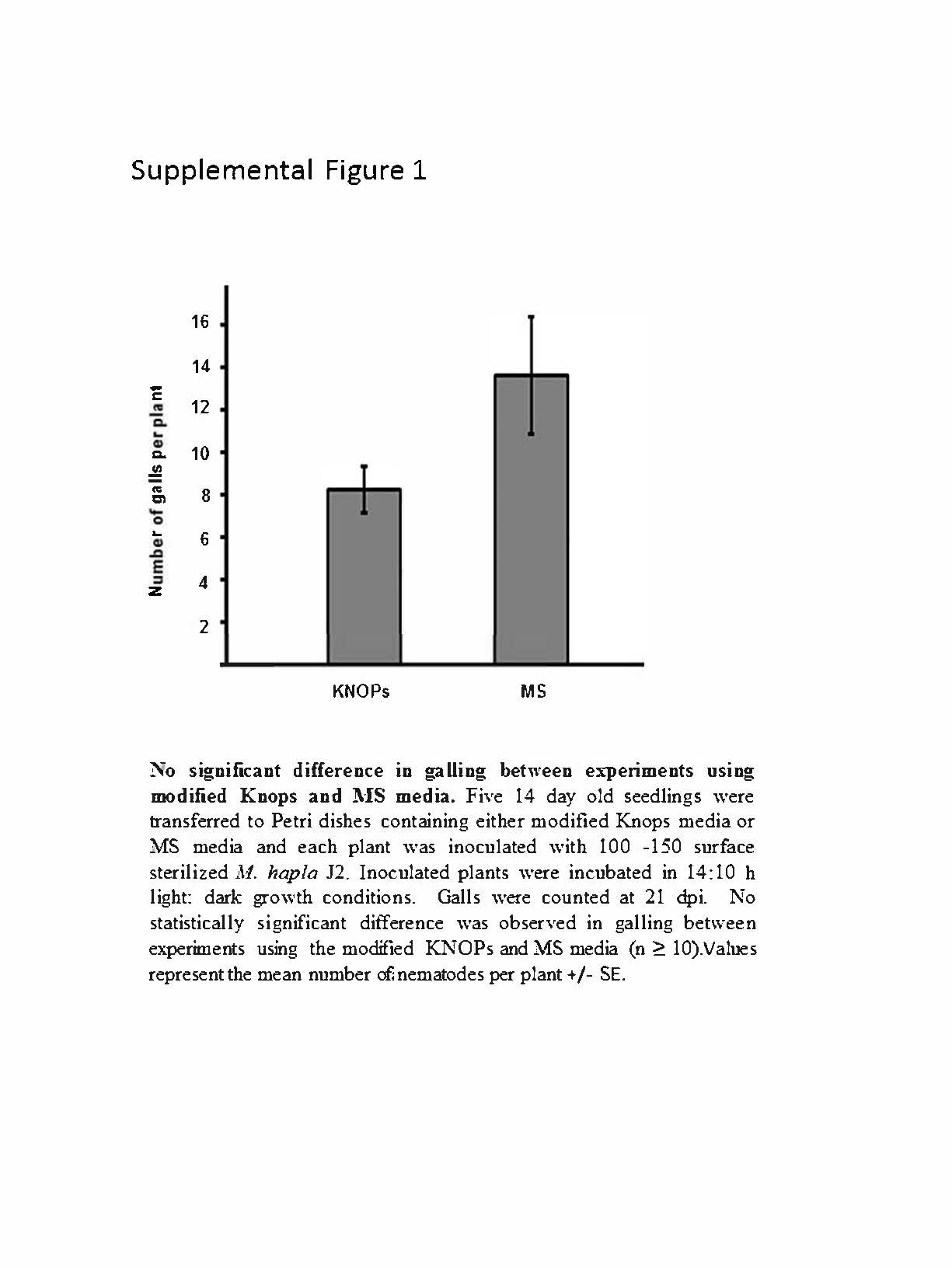

Supplement: Supplementary file 1 [file Image_1.jpg]

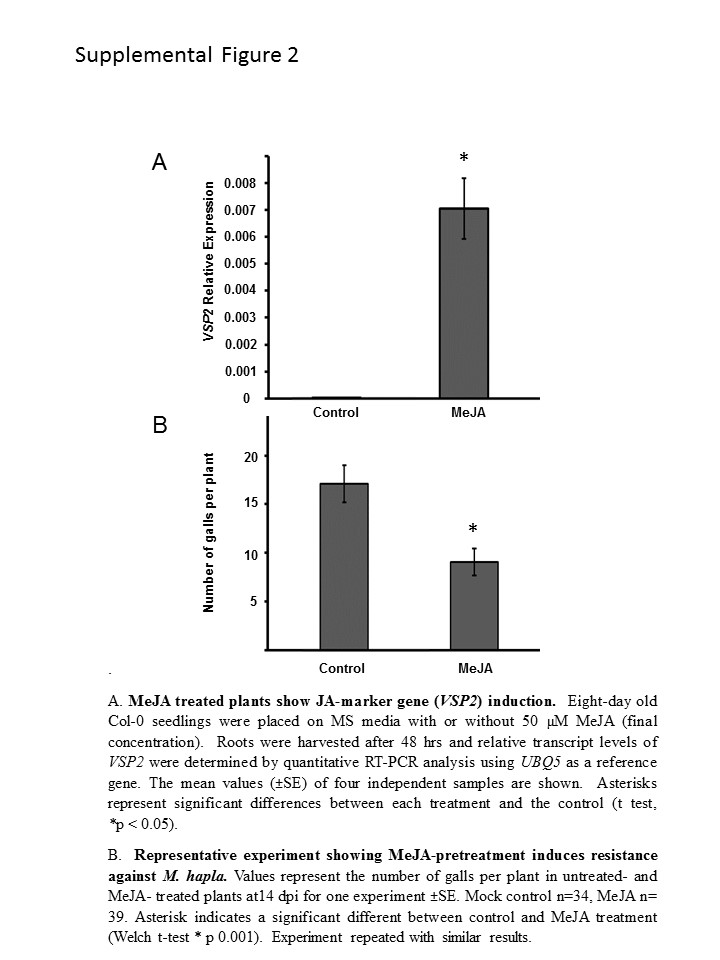

Supplement: Supplementary file 2 [file Image_2.JPEG]

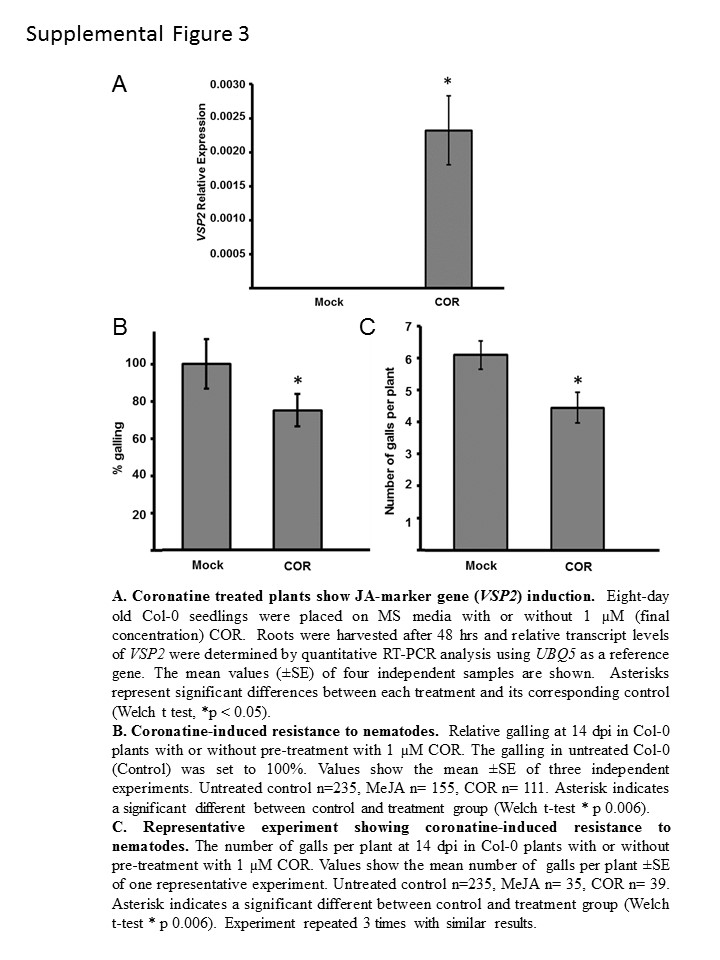

Supplement: Supplementary file 3 [file Image_3.JPEG]

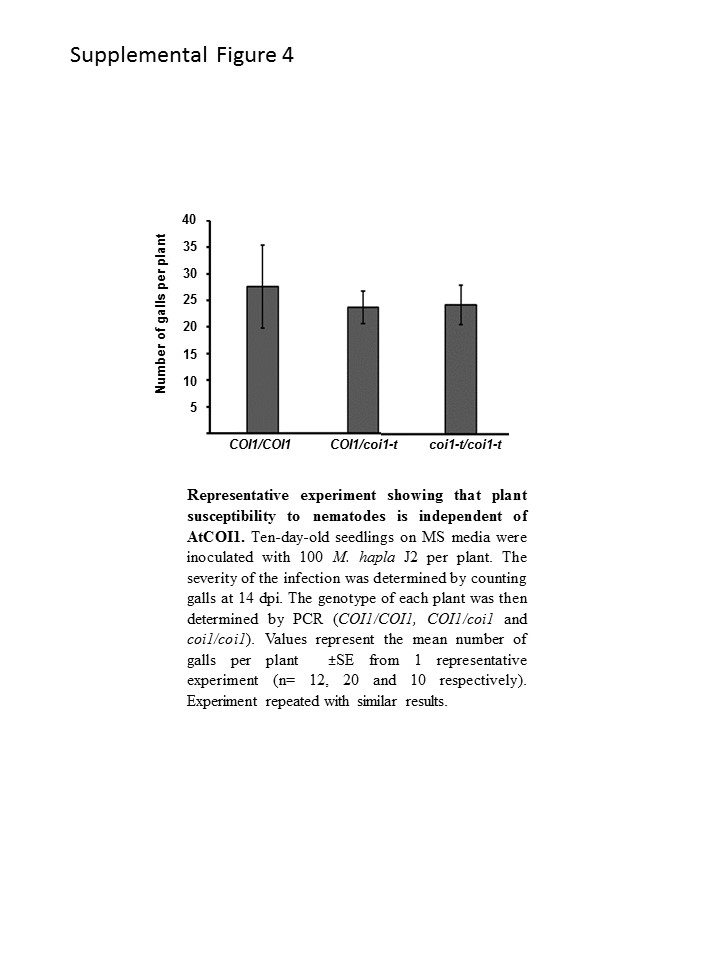

Supplement: Supplementary file 4 [file Image_4.JPEG]

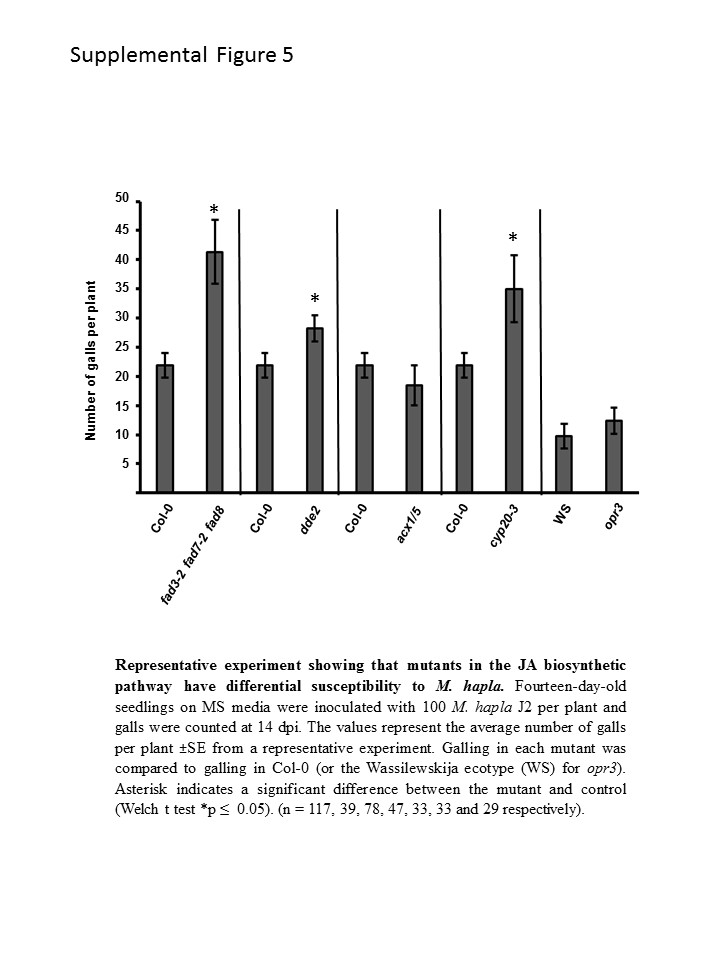

Supplement: Supplementary file 5 [file Image_5.JPEG]

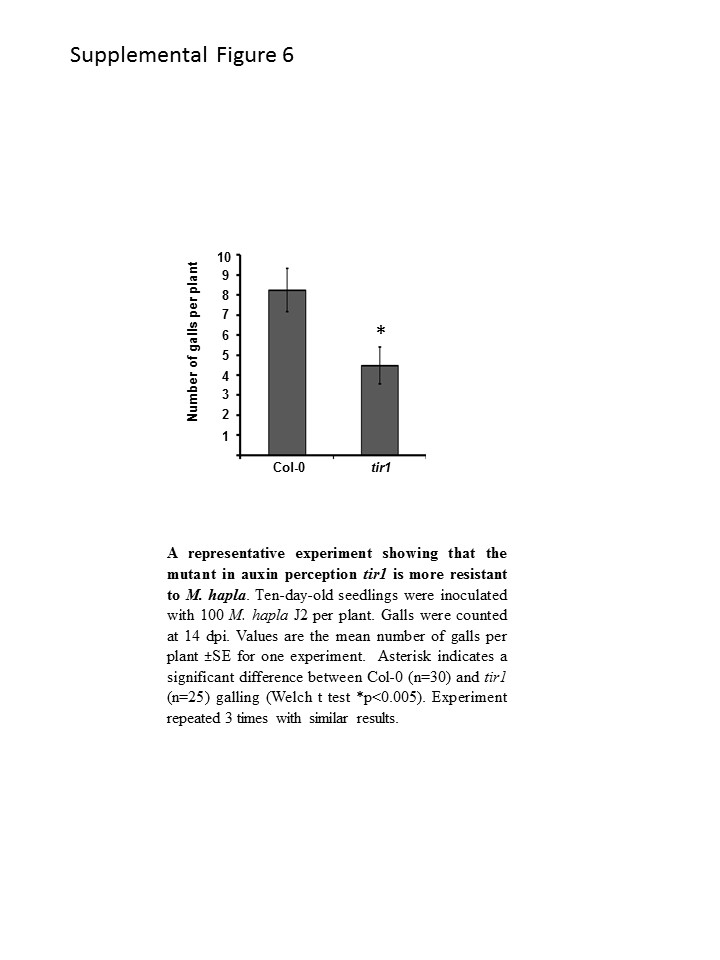

Supplement: Supplementary file 6 [file Image_6.JPEG]
